# Can mechanical load and physiological intensity during gymnastics training explain physical adaptations? an observational study

**DOI:** 10.3389/fphys.2025.1645514

**Published:** 2025-08-18

**Authors:** Ying Zhou, Liuxi Yang

**Affiliations:** ^1^ Physical Education of Sichuan Normal University, Chengdu, Sichuan, China; ^2^ Civil Aviation Security College, Civil Aviation Flight University of China, Guanghan, China

**Keywords:** artistic gymnasts, training load monitoring, physical adaptations, strength, aerobic capacity, youth athletes

## Abstract

This study analyzed the relationships between physiological and mechanical training loads and subsequent physical strength and aerobic adaptations in youth gymnasts. A prospective cohort design monitored 40 local-level female artistic gymnasts (16.5 ± 1.1 years) over a 12-week preparatory training phase. Strength (Isometric Mid-Thigh Pull [IMTP], Countermovement Jump [CMJ]) and aerobic fitness (Multistage Aerobic Fitness Test by Luc Léger [MSAFT]) were assessed at baseline and post-intervention. Training load was continuously monitored via heart rate (TRIMP), mechanical load (jumps), and ratings of perceived exertion (RPE). Significant improvements were observed across all fitness parameters: IMTP (19.2%), CMJ (2.7%), and MSAFT (8.6%), all with large effect sizes (p < 0.001). Mean session loads averaged RPE 7.25 ± 0.732, TRIMP 290.45 ± 30.343, and 105.18 ± 27.547 jumps. Jump volume significantly correlated with improvements in IMTP (r = 0.478, p = 0.002) and CMJ (r = 0.785, p < 0.001), indicating its large association with strength improvement. Conversely, RPE (r = 0.775, p < 0.001) and TRIMP (r = 0.872, p < 0.001) were largely correlated with enhanced aerobic fitness. This study showed that physiological training loads are important for aerobic adaptations, while mechanical loads, particularly jumps, drive strength and power improvements in youth gymnasts. Therefore, monitoring strategies that integrate both internal and external load parameters is essential for optimizing specific physical qualities in gymnasts.

## 1 Introduction

The concept of training load (TL) has been widely used in sports science monitoring practices. TL is conceptualized as a multidimensional construct with internal and external subdimensions (Impellizzeri et al., 2019). External load typically refers to measurable physical demands like distance, speed, and jumps, while internal load encompasses physiological and psychological responses such as heart rate and perceived exertion (Bourdon et al., 2017; Williams et al., 2017). However, it is accepted that integrating both external and internal load monitoring provides a more comprehensive understanding of athletes’ responses to training and competition (Vanrenterghem et al., 2017).

The state of the art in training load monitoring has become well-established (McGuigan, 2017) over the past decade, particularly following the introduction of technologies such as inertial measurement units (IMUs), global positioning systems (GPS), and other tools for assessing the physical demands placed on athletes in both training and competitive settings (West et al., 2021). Research in this area has been especially robust in characterizing physical demands within training contexts, describing periodization patterns and variations across and within weeks (Freitas et al., 2025), analyzing inter-athlete variability under similar conditions, and investigating associations between training load and injury risk (Leupold et al., 2025). Additionally, studies have explored whether the magnitude of training load is linked to physiological and performance adaptations in athletes (Borresen and Ian Lambert, 2009).

Research on the dose-response relationship between training load and aerobic fitness improvements in athletes shows mixed results. Some studies found significant correlations between certain training load measures and fitness changes. For example, Banister’s TRIMP explained 78% of variance in VO2max changes in rugby players (Taylor et al., 2018). Similarly, time spent above maximal aerobic speed showed a very large association with aerobic fitness improvements in youth soccer players (Fitzpatrick et al., 2018). However, other research found no linear dose-response relationship between common training load measures and performance improvements in recreational cyclists (Vermeire et al., 2021). The discrepancies may be due to differences in athlete populations and training load quantification methods. There is no universally accepted “gold standard” for measuring training load across sports (Lambert and Borresen, 2010). Some evidence suggests that training intensity distribution might be a key factor in establishing relationships with performance improvements (Vermeire et al., 2021).

Despite the rapid growth of research on training load in various team and individual sports, studies focusing on gymnasts remain scarce and limited. Research on collegiate women’s gymnastics found fluctuations in acute:chronic workload ratio and weekly training load throughout the season, with positive correlations between wellness variables and training load measures (Leupold et al., 2025). In elite youth women’s artistic gymnastics, variations in external and internal training loads were observed across microcycles, with significant correlations between weekly internal load and total elements performed (Freitas et al., 2025). Heart rate monitoring in Chinese gymnasts showed brief peaks exceeding 190 beats per minute during training, emphasizing the high-intensity nature of the sport (Zhang, 2022). The training load and recovery profiles of professional rhythmic gymnasts across a season were characterized, showing changes during competitive periods (Debien et al., 2020).

While the above mentioned studies have begun to explore training load patterns in gymnasts—such as fluctuations in internal and external loads across training cycles (Freitas et al., 2025) and correlations with wellness or performance indicators (Freitas et al., 2025) — none have directly examined how these loads translate into measurable physical adaptations, particularly in terms of strength and aerobic development. In other words, research in gymnastics has largely focused on describing training load, rather than investigating how these loads are associated with specific physical adaptations. This represents a gap in the literature when compared to other sports. Most existing research has focused on load characterization or short-term physiological responses rather than long-term outcomes. Consequently, a critical gap remains in understanding the dose-response relationship between specific training load components and physical strength adaptations in gymnastics. Given the sport’s high-intensity efforts, technical demands, and early specialization, investigating how different dimensions of training load contribute to strength gains can be essential. Therefore, the aim of this study is to analyze the relationships between perceived exertion, heart rate training impulse, and number of jumps, and their associations with countermovement jump, isometric mid-thigh pull, and multistage aerobic fitness adaptations in youth gymnasts. Additionally, the study seeks to identify the determinants of effort intensity contributing to these adaptations.

## 2 Methods

### 2.1 Study design and setting

This study employed a prospective cohort design to assess gymnasts’ strength levels at two key points: baseline and following 12 weeks of consistent training. Throughout the entire training period, detailed monitoring of training load was also conducted. Given its observational nature, the research team maintained no influence over the gymnasts’ training plans or interventions, serving solely in evaluative and monitoring capacities. To mitigate potential analysis bias, the research personnel responsible for measuring strength levels at baseline and post-12 weeks were different from those involved in monitoring training load, and were also blinded to participant identities during these assessments. The study coincided with the initial phase of the gymnastics season—the preparation phase—characterized by an emphasis on physical conditioning, the development of individual skills, and the acquisition of new routines. On average, these athletes trained six times per week and competed at a local level. The recruitment process began with invitations extended to local gymnastics clubs and their directors, who subsequently invited parents and eligible gymnasts to participate as volunteers.

### 2.2 Participants

We established participant inclusion criteria *a priori*. Participants had to be female artistic gymnasts aged between 15 and 18 years old, possess more than 3 years of experience, and be free from injury or any health condition throughout the study period and for the month before its start. Additionally, they could not miss any strength assessment time points, had to attend at least 90% of their club’s scheduled training sessions, and could not be taking any drugs or other substances that might affect performance.

After recruiting from two local clubs, we identified 46 potential participants who expressed willingness to join. However, we excluded four who were injured at the beginning of the study, and two others became injured during the observational period. This reduced our final sample to 40 female artistic gymnasts competing at a local level. On average, they were 16.5 ± 1.1 years old, had 4.5 ± 1.1 years of experience, a height of 149.7 ± 3.8 cm, a body mass of 43.4 ± 2.9 kg, and a body mass index (BMI) of 19.4 ± 0.3 kg/m^2^. These participants typically trained at least six times a week, with session durations varying from 120 to 140 min depending on the specific training day.

Over the 12-week preparation phase, the gymnasts followed their respective club’s established training programsEach weekly microcycle consisted of approximately six training sessions, each lasting between 120 and 140 min. Sessions were generally structured into three main phases: a 20–30 min warm-up emphasizing mobility and general conditioning, a 70–90 min main training block focused on strength, technical skill development, and apparatus-specific drills, followed by a 20–30 min cooldown and flexibility work.

The focus of training varied systematically across the week to balance physical conditioning with skill acquisition and routine refinement. Early-week sessions prioritized general and specific strength training, including plyometrics, core stability, and resistance exercises targeting gymnastics-relevant muscle groups. Midweek sessions concentrated more heavily on technical skill drills and apparatus-specific practice, such as vault approach mechanics, uneven bars swing techniques, balance beam stability, and floor exercise choreography. Towards the end of the week, sessions typically emphasized routine integration, combining skills into full routines with attention to execution quality and endurance. Flexibility and injury prevention exercises were incorporated throughout the week, with particular emphasis during cooldowns.

Although the overall weekly structure was consistent, variations existed between clubs and individual coaches regarding the exact exercises, skill progression, and volume emphasis. No uniform training program was imposed by the research team, reflecting the observational nature of the study. This coach-specific variability in training content and emphasis is acknowledged as a factor potentially influencing adaptation responses independent of total training load, which was monitored.

The university ethical committee (Sichuan Normal University; code 2025LS0040) gave preliminary approval for the study, which followed all ethical procedures. This included informing participants and their parents about the study’s design, risks, and benefits. We also obtained informed consent, with parents signing on behalf of their daughters. We fully informed them that participation in the study was voluntary and that they could withdraw at any time without any consequences.

### 2.3 Physical measurements and procedures

Assessments were conducted at the first training session of the week, after a 24-h rest period following the last session. The assessments took place in the afternoon, around 4 p.m., beginning with the collection of anthropometric data. The assessment period was limited by participant availability. Although this could have affected the anthropometric data, it was not considered in the main analysis of the study. Afterward, the gymnasts performed a specific warm-up routine, consisting of light jogging followed by 15 min of dynamic stretching and jumps, aimed at preparing them for the assessments. The assessments were carried out in the club’s indoor facilities, where the temperature was maintained between 20 °C and 22 °C and relative humidity between 50% and 60%. After the warm-up, a 3-min rest was provided. The gymnasts were then evaluated in the same sequence: first performing the isometric mid-thigh pull test, followed by a 3-min rest, then the countermovement jump test, and finally the multistage aerobic fitness test.

#### 2.3.1 Isometric mid-thigh pull test (IMTP)

To assess isometric mid-thigh pull (IMTP) performance, we had gymnasts perform the test using a crane scale. This specific crane scale (CS, with a capacity of 300 kg (kg) and measuring in 0.1 kg increments) was securely mounted to a rigid, immovable frame. This setup was adopted following the recommendations of the original validation study for this procedure (Urquhart et al., 2018), ensuring consistency and reliability in our measurements. Before each repetition, athletes were instructed to assume a standardized position: feet positioned shoulder-width apart directly beneath the crane scale, knees flexed to approximately 135°, and hips slightly abducted. The reference angle was measured using a goniometer during the familiarization trial for each athlete. Although this measurement was not repeated during data collection, the familiarization session helped ensure consistent positioning, allowing only for natural slight variations. The gymnasts grasped a padded bar attached to the crane scale with an overhand, pronated grip, ensuring their arms were fully extended. Verbal instructions emphasized generating maximal concentric force as quickly as possible, producing maximal upward force as if performing a full extension. Each pull was sustained for a duration of 5 s, with strong verbal encouragement provided throughout. Besides one familiarization trial, three maximal effort repetitions were performed, with a 2-min rest period between attempts. It is also important to note that the athletes were already familiar with the test, as it is part of their regular assessment routine. The highest peak force, standardized to body mass (expressed in Newtons/kilogram), achieved across all valid trials was recorded and subsequently used for analysis. The coefficient of variation, expressed as a percentage, was calculated to assess variation within the gymnasts, showing an average of 4.1% ± 0.3% across the evaluation sessions.

#### 2.3.2 Countermovement jump test (CMJ)

To assess countermovement jump (CMJ) performance, gymnasts executed the test using the ChronoJump mat system. This system (ChronoJump Boscosystems, Barcelona, Spain) utilizes a specialized jump mat to automatically calculate jump height based on flight time. Previous research has showed the reliability and validity of this system for measuring CMJ performance (Pueo et al., 2020). For each jump, the athlete was instructed to stand upright with hands on hips (to eliminate arm swing), then perform a rapid downward movement by flexing the knees and hips to approximately 90° (a reference angle previously familiarized using a goniometer), immediately followed by a maximal vertical jump. The ChronoJump system, connected to a computer running the ChronoJump software, registered the moments of take-off and landing. Besides one familiarization trial, three maximal effort repetitions were performed, with a 30-s rest period between attempts. The highest jump height (in centimeters) achieved across the valid trials was recorded and used for subsequent analysis. To evaluate the variability within gymnasts, the coefficient of variation was determined as a percentage, with a mean value of 3.2% ± 0.4% observed throughout the assessment sessions.

#### 2.3.3 Multistage aerobic fitness test (MSAFT)

To evaluate aerobic fitness, gymnasts underwent the 20-m Multistage Aerobic Fitness Test, commonly known as the Luc Léger test or beep test (Mayorga-Vega et al., 2015). This standardized field test involved continuous shuttle running between two lines set 20 m apart, in time with pre-recorded audio signals. Participants began the test with an initial speed of 8.5 km/h. The test is structured into 1-min stages, and at the end of each stage (signaled by a double beep), the required running speed progressively increases by 0.5 km/h. Participants were instructed to reach the 20-m line with each single beep. The test continued until the gymnast was unable to reach the 20-m line in synchronization with the beep for two consecutive occasions, or until volitional exhaustion. The total distance covered (in meters), representing the last completed shuttle, was recorded as each gymnast’s score.

### 2.4 Load monitoring

For all scheduled training sessions, the research team continuously monitored the gymnasts’ training load. This involved tracking heart rate (HR) and mechanical load throughout each session. Additionally, ratings of perceived exertion (RPE) were collected from each gymnast approximately 30 min after the session concluded. This post-session RPE aimed to quantify their subjective intensity experience.

#### 2.4.1 Rate of perceived exertion

To quantify the subjective experience of exertion after gymnastic training sessions, the Borg Category Ratio 10 (CR-10) Scale was implemented. This scale is designed to measure the intensity of perceived exertion. Before any testing, all gymnasts underwent a thorough familiarization process with the Borg CR-10 scale. This involved an explanation of the scale’s purpose and its numerical and verbal anchors. Participants were instructed to rate their perceived effort using the corresponding numbers and verbal descriptors 30 min after completing the training session. This timing follows a previous studies, including one (Christen et al., 2016) that found post-exercise timing does not significantly affect session RPE after either steady-state or interval exercise. The primary verbal anchors used were: 0 (Nothing at all), 1 (Very weak), 2 (Weak - light), 3 (Moderate), 4 (Somewhat strong), 5 (Strong - heavy), 7 (Very strong), and 10 (Extremely strong - Maximal). Participants were explicitly informed that they could use intermediate values (e.g., 2.5, 4.5) to reflect their perceived effort if it fell between two described points. This familiarization ensured a common understanding of the scale and its application across all participants, aiming to enhance the reliability of their ratings. To minimize any potential influence from other participants, RPE were individually recorded on a specially designed sheet. Participants provided their answers privately to a research team member.

#### 2.4.2 Heart rate monitoring

HR was continuously monitored throughout all training sessions using Polar H10 Bluetooth heart rate sensors (Polar Electro Oy, Kempele, Finland). Each participant wore a chest strap with the H10 sensor. The real-time heart rate data from all participants was simultaneously tracked and displayed using the Polar Team application (Polar Electro Oy, Kempele, Finland) on a dedicated tablet, allowing for live monitoring. To standardize the intensity of training and assess internal training load, heart rate data was normalized to each individual gymnast’s maximal heart rate (HRmax), which was determined during the preceding 20-m Multistage Aerobic Fitness Test. This approach is justified, as a previous study found no difference in maximal heart rate between treadmill tests and the multistage test (Selland et al., 2022). The outcome extracted from the heart rate data was Training Impulse (TRIMP), calculated using the Edwards method. This method quantifies training load by assigning different weighting factors to time spent within specific heart rate zones relative to HRmax: Zone 1 (50%–60% HRmax), Zone 2 (60%–70% HRmax), Zone 3 (70%–80% HRmax), Zone 4 (80%–90% HRmax), and Zone 5 (90%–100% HRmax). The duration (in minutes) spent in each zone was multiplied by its corresponding weighting factor (e.g., 1 for Zone 1, 2 for Zone 2, etc.), and these products were summed to yield a total TRIMP score for each session.

#### 2.4.3 Mechanical load

To quantify mechanical load during training sessions, specifically the number of jumps, the My Vert IMU system was utilized. This involved equipping each gymnast with a small, lightweight Inertial Measurement Unit (IMU) sensor (worn on the hip to capture whole-body movement). The IMU, which integrates a tri-axial accelerometer and gyroscope, continuously recorded raw acceleration and angular velocity data throughout the session. The My Vert application automatically identify and count each discrete jump by detecting characteristic patterns in the vertical acceleration signal associated with the take-off and landing phases (Benson et al., 2020). The system recognizes the instances when the gymnast’s feet leave and return to the ground. This automated detection provided a count of the total number of jumps performed by each gymnast during every training session.

### 2.5 Statistical procedures

We primarily aimed to investigate the association between internal and external training load measures and changes in physical fitness parameters over the 12-week intervention period. To achieve this, we performed a correlational analysis. Our independent variables, representing training load, were the mean RPE, mean TRIMP, and mean number of jumps, all averaged across the 12 weeks of training. The dependent variables, representing changes in physical fitness, were the delta values of the CMJ height, MTP peak force, and 20-m Multistage Aerobic Fitness Test distance. We calculated delta values (Δ) for CMJ, IMTP, and the 20-m Multistage Aerobic Fitness Test by subtracting each individual’s baseline (pre-intervention) measurement from their post-intervention measurement. This approach allowed us to assess the absolute change in each fitness parameter over the 12-week period.

We used Pearson product-moment correlation coefficients (r) to assess the strength and direction of the linear relationships between the mean training load variables and the delta values of physical fitness. Additionally, we employed paired samples t-tests to compare pre- and post-intervention measures for relevant variables. For the paired samples t-tests, we calculated Cohen’s d to determine the effect size, with values of 0.2, 0.5, and 0.8 considered as small, medium, and large effects, respectively.

Before conducting these analyses, we tested assumptions for each pair of variables. We assessed the normality of each variable’s distribution using the Shapiro-Wilk test, along with visual inspection of Q-Q plots and histograms. We also assessed the assumptions of linearity and homoscedasticity (equal variance of residuals across the range of predicted values) by visually inspecting scatterplots. We applied Pearson correlations and paired t-tests after assessing these assumptions. To interpret the magnitude of the correlations, we considered r thresholds of 0.1, 0.3, and 0.5 as small, medium, and large effects, respectively (Cohen, 1988). The significance level for all statistical tests was set at α = 0.05. All statistical analyses were conducted using JASP (Version 0.19.3).

## 3 Results

Table 1 presents the descriptive statistics for the IMTP, CMJ, and Multistage Aerobic Fitness Test, showing pre- and post-intervention means, standard deviations, mean differences, and percentage changes for the 40 participants. A statistically significant increase was found for the IMTP, with post-intervention scores being higher than pre-intervention scores, t(39) = 20.391, p < 0.001. The effect size for IMTP was large (Cohen′s d = 3.224). Similarly, CMJ scores significantly increased from pre-to post-intervention, t(39) = 8.138, p < 0.001, indicating a large effect (Cohen′s d = 1.287). Finally, a significant improvement was observed in the Multistage Aerobic Fitness Test scores from pre-to post-intervention, t(39) = 12.183, p < 0.001, also representing a large effect (Cohen′s d = 1.926).

**TABLE 1:** Descriptive statistics for pre and post-intervention tests (N = 40).

Test	Mean (Pre)	SD (Pre)	Mean (Post)	SD (Post)	Mean difference (Post-Pre)	Percentage change
IMTP (N/kg)	21.6	5.2	25.7	6.4	4.1	19.2%
CMJ (cm)	31.4	1.9	32.2	1.5	1.2	2.7%
MSAFT (m)	1,310.0	9.3	1,422.0	9.9	112.0	8.6%

IMTP: Isometric mid-thigh pull test; CMJ: countermovement jump test; MSAFT: multistage aerobic fitness test; Percentage Change is calculated as ((Mean Post - Mean Pre)/Mean Pre) * 100.

Descriptive statistics for the load variables for session revealed an average MeanRPE of 7.25 (standard-deviation, [SD] = 0.732), indicating a perceived exertion generally above moderate intensity, with a tight range from 6.0 to 8.5. For MeanTRIMP, the average score was 290.45 (SD = 30.343), varying from a minimum of 240 to a maximum of 342. Lastly, MeanJumps had an average of 105.18 (SD = 27.547), with individual values spanning from 60 to 150 jumps.

**FIGURE 1:**
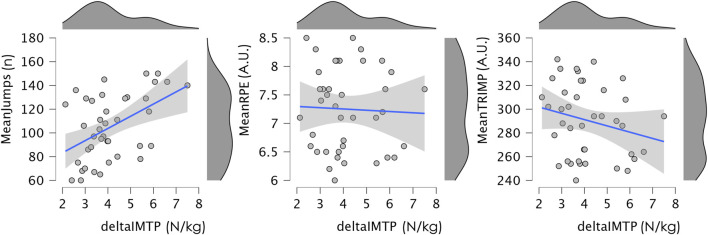
Scatter plot illustrating the relationship between deltaIMTP and MeanJumps, meanRPE and meanTRIMP, with a linear regression line and 95% confidence interval. Marginal density plots for each variable are also shown. IMTP: isometric mid-thigh pull test; RPE: ratings of perceived exertion; TRIMP: training impulse.

A statistically significant positive correlation was observed between MeanJumps and deltaIMTP (r = 0.478, 95% CI [0.190, 0.684], p = 0.002), indicating a moderate association suggesting that higher jump volume was related to greater improvements in IMTP. However, no significant correlations were found between deltaIMTP and MeanRPE (r = −0.039, 95% CI [−0.346, 0.276], p = 0.810) or MeanTRIMP (r = −0.224, 95% CI [−0.498, 0.097], p = 0.165). Figure 1 illustrates the relationships between deltaIMTP and MeanJumps, meanRPE and meanTRIMP.

**FIGURE 2:**
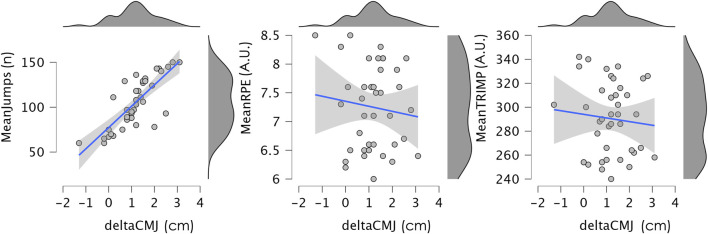
Scatter plot illustrating the relationship between deltaCMJ and MeanJumps, meanRPE and meanTRIMP, with a linear regression line and 95% confidence interval. Marginal density plots for each variable are also shown. CMJ: countermovement jump; RPE: ratings of perceived exertion; TRIMP: training impulse.

For deltaCMJ, a large positive correlation was found with MeanJumps (r = 0.785, 95% CI [0.620, 0.879], p < 0.001), indicating that higher jump volumes were largely related to greater improvements in CMJ performance. No significant correlations were observed between deltaCMJ and MeanRPE (r = −0.109, 95% CI [−0.406, 0.211], p = 0.502) or MeanTRIMP (r = −0.091, 95% CI [−0.390, 0.228], p = 0.577). Figure 2 illustrates the relationships between deltaCMJ and MeanJumps, meanRPE and meanTRIMP.

**FIGURE 3:**
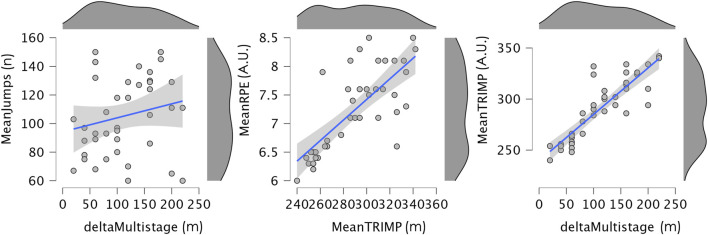
Scatter plot illustrating the relationship between deltamultistage and MeanJumps, meanRPE and meanTRIMP, with a linear regression line and 95% confidence interval. Marginal density plots for each variable are also shown. RPE: ratings of perceived exertion; TRIMP: training impulse.

Regarding the Multistage Aerobic Fitness Test, deltaMSAFT was largely and positively correlated with MeanRPE (r = 0.775, 95% CI [0.605,0.873], p < 0.001), indicating a large association, and MeanTRIMP (r = 0.872, 95% CI [0.765,0.929], p < 0.001), also showing a large association. These findings suggest that higher perceived exertion and training impulse were largely associated with greater improvements in aerobic fitness. There was no significant correlation between deltaMultistage and MeanJumps (r = 0.206, 95% CI [−0.115,0.485], p = 0.201). Figure 3 illustrates the relationships between deltaMSAFT and MeanJumps, meanRPE and meanTRIMP.

## 4 Discussion

The present study aimed to explore the specific relationships between physiological and mechanical training loads and subsequent physical adaptations in youth artistic gymnasts. Our findings reveal significant improvements in both strength (IMTP and CMJ) and aerobic capacity (MSAFT) over a 12-week preparatory phase, showing a load-specificity in these adaptations. Specifically, while enhancements in neuromuscular strength were largely correlated with the volume of mechanical load (jumps), improvements in aerobic capacity were largely associated with greater physiological stimuli, as indicated by RPE and TRIMP.

Regarding IMTP performance, our findings showed a significant increase in strength, accompanied by a positive correlation with the volume jumps during training, yet no significant association with physiological load parameters (RPE and TRIMP). This suggests that improvements in maximal isometric strength in youth gymnasts are highly associated with the repeated application of explosive, high-force mechanical stimuli (Dotan et al., 2013). Possibly, the number of jumps can ultimately target motor unit recruitment, increased neural drive, and improved rate of force development stemming from the rapid, powerful contractions inherent in jumping activities (Ramirez-Campillo et al., 2021). Repeated exposure to such demands may facilitates specific adaptations within the force-velocity continuum, pushing the capabilities for maximal force output (Samozino, 2018). Conversely, the lack of correlation with physiological load measures like RPE and TRIMP indicates that these markers, while reflecting overall internal stress and metabolic demand (Scott et al., 2016), may not be sufficiently sensitive or specific to quantify the localized mechanical tension and neural drive primarily responsible for maximal strength gains.

Further extending our analysis of strength adaptations, the CMJ performance also showed significant improvement, exhibiting an even larger positive correlation with the cumulative jump volume during training sessions, while again showing no significant association with RPE or TRIMP. This relationship accentuates a possible specificity of training required for explosive power development (Markovic et al., 2013). The direct and repeated exposure to the stretch-shortening cycle during numerous jumps eventually is associated with neuromuscular adaptations, including increased firing frequency, and improved utilization of elastic energy, all important to superior CMJ performance (Markovic and Mikulic, 2010). The biomechanical congruence between the training stimulus (various jumps) and the assessment (CMJ) likely accounts for this particularly large correlation. Conversely, the absence of a link between CMJ improvements and physiological load measures suggests that maximal power improvements are less related with the overall metabolic or cardiovascular stress (Douglas et al., 2021) captured by RPE and TRIMP.

In the context of aerobic fitness, our analysis revealed a significant improvement in MSAFT performance, which was largely and positively correlated with both MeanRPE and MeanTRIMP. Conversely, no significant association was found between MSAFT improvements and the mechanical load quantified by jump volume. This large relationship suggests that improvements in aerobic capacity in youth gymnasts can be associated with the accumulation of physiological stimulus (Jaspers et al., 2017). RPE and TRIMP, likely capture the integrated cardiovascular and metabolic demands (Borresen and Lambert, 2008) that are paramount for inducing aerobic adaptations such as enhanced oxygen transport and utilization, increased mitochondrial density, and improved cardiorespiratory efficiency (Rivera-Brown and Frontera, 2012). The absence of a relationship with jump volume may indicate the specificity of energy system adaptations, indicating that while explosive mechanical work contributes to strength and power, it does not provide the sustained metabolic stress necessary to significantly enhance aerobic endurance.

Despite our findings on load-adaptation relationships, this study is not without limitations. Primarily, its observational design, while ecologically valid, precludes establishing definitive cause-and-effect relationships or controlling for specific training interventions, thus limiting the generalizability of direct dose-response conclusions. Future research should consider employing randomized controlled trials to systematically manipulate training loads and directly assess their causal impact on specific adaptations across diverse populations of gymnasts. Furthermore, while RPE, TRIMP, and jump volume provided essential insights into training load, the multifaceted nature of gymnastics suggests that future research could benefit from more comprehensive monitoring methods. These may include direct measurements of impact forces during landings or detailed assessments of time under tension during strength elements. Additional data on mechanical loading, particularly ground reaction forces, would offer valuable insight into the relationships between training load and performance improvements.

Although peak height velocity (PHV) was not directly analyzed in the female gymnasts, the limited age range of 15–18 years significantly minimizes the confounding effect of maturation, as existing evidence indicates that PHV in female athletes, including those from Chinese cohorts, typically occurs well before age 15. For instance, a longitudinal study of Chinese girls aged ∼9.5–10.5 years at baseline reports that PHV occurred approximately 0–3 years before menarche, with the mean age of menarche at 12.1 ± 1.0 years (Zhu et al., 2008). Therefore, it is reasonable to assume that all participants had reached or surpassed their PHV, placing them in a post-maturational stage and reducing variability in growth-related physical adaptations during the observation period. Another possible confounding factor was the menstrual cycle, which was not analyzed but could influence adaptations. Future research should take this into consideration.

Although the limitations, in practice, our findings stress that optimizing physical development in youth gymnasts demands a specific approach to training load monitoring. Coaches should integrate both physiological (e.g., RPE, TRIMP) and mechanical (e.g., jump volume) load measures to control internal and external stimulus and the corresponding aerobic improvements and strength/power improvements, respectively. This may help to align training with desired adaptations, moving beyond a one-size-fits-all training and monitoring strategy.

## 5 Conclusion

This study delivers evidence for the load-specificity of adaptations in youth artistic gymnasts. Our findings show that greater physiological stimulus, as quantified by TRIMP and RPE, is largely related to enhanced aerobic capacity. Conversely, improvements in neuromuscular strength and power, evidenced by improvements in IMTP and CMJ performance, are primarily associated with the volume of mechanical load, specifically the number of jumps performed during training sessions. These different relationships emphasize the importance of adopting a multidimensional and non-unique monitoring process for gymnasts. Relying solely on physiological markers might overlook the mechanical stimuli crucial for strength development, while focusing only on mechanical loads could obscure the physiological demands driving aerobic improvements. Therefore, coaches and practitioners may implement monitoring strategies that simultaneously track both internal (physiological, e.g., RPE, TRIMP) and external (mechanical, e.g., jump volume) training loads. This integrated approach allows for a more complete understanding of how specific training stimuli contribute to aimed physical adaptations, ultimately enabling optimized program design.

## Data Availability

The raw data supporting the conclusions of this article will be made available by the authors, without undue reservation.
